# Residual Stress and Microstructure Characterization of 34CrMo4 Steel Modified by Shot Peening

**DOI:** 10.1155/2020/5367345

**Published:** 2020-03-11

**Authors:** Kejian Li, Xu Wu, Liping Chen, Dengming Chen, Gungjun Zhu, Qian Shen, Jae Hong Yoon

**Affiliations:** ^1^School of Metallurgy and Materials Engineering, Chongqing University of Science & Technology, Chongqing 401331, China; ^2^College of Material Science and Engineering, Chongqing University of Technology, Chongqing 400054, China; ^3^School of Nano & Advanced Materials Engineering, Changwon National University, Changwon 51140, Republic of Korea

## Abstract

34CrMo4 steel is widely used for drill stem in oil exploration, because of its excellent properties, such as favorable hardenability, shock absorption, less tendency of temper brittleness, and eminent wear resistance. In this study, the main works are residual stress test and microstructure characterization of 34CrMo4 steel upon various shot peening treatments. The residual stress distribution with effect depth was studied upon the shot peening. Face-to-face paste sample preparation method is required for continuous observation for microstructure evolution of shot-peened specimen from the treat surface to matrix. Grain refinement, lath structure, and precipitates are clearly observed in the gradient deformation layer.

## 1. Introduction

34CrMo4 steel is one of the representative medium-carbon and low-alloy steels, due to its good balance of strength, toughness, and wear resistance in an extreme working environment. Hence, 34CrMo4 steel is widely used for drill stem in oil exploration, because of its excellent properties, such as favorable hardenability, shock absorption, less tendency of temper brittleness, and good corrosion resistance [1].

Surface treatment is widely used in increasing the performance of materials, such as elements coating to protect the material from severe environments [2]. The effect of hot deformation parameters such as temperature and strain rate on dynamic restoration processes of 34CrMo4 steel was studied in previous research [3].

Severe plastic deformation (SPD), such as shot peening [4, 5], is a generic term describing a group of metalworking techniques. It includes very large strains and typically involves a complex stress state or high shear, resulting in a high defect density and equiaxed ultrafine grain size (diameter less than 500 nm), even nanocrystalline structure (diameter less than 100 nm). The inference depth is about 200~600 *μ*m after shot peening surface treatment. The microstructure will deform to include cracks, laths, lattice distortion, disordered structures, and deformation grains [6–8]. For the 34CrMo4 steel tools, after a long period of service, the purpose of this experiment is try to remove the rust and repair the 34CrMo4 steel tools by shot peening treatment. Shot peening is a well-known cold surface treatment that consists in bombarding the surface of the component with a stream of shots. As a result, the compressive residual stress and microstructure modification are induced within the near surface, which can improve the fatigue performance and surface properties of the material [9–11]. Therefore, the microstructure evolution analysis of the deformation layer from the top surface to matrix is very important.

Electron microscopy (EM) comprises a wide range of different methods that use various signals arising from the interaction of an electron beam with the sample to obtain information about the structure, morphology, and composition. Along with the development of materials science research on the basis of EM, scanning electron microscopy (SEM) and transmission electron microscopy (TEM) are widely used in the characterization of metals, ceramics, powder, biology, and composites [12, 13]. With high-resolution electron microscopy (HREM) analysis technology, a resolution below 0.2 nm can reveal to us the essence of material at the atomic level. At the same time, ultra-high resolution microscopic analysis techniques for TEM sample preparation are becoming increasingly necessary to meet a higher quality. The traditional metallographic polishing, double ion milling, electrolytic polishing, and electrolytic jet are becoming increasingly unsuitable to meet the standards of scientific research methods [14, 15]. In recent years, because of the advent and development of focused ion beam (FIB) instruments, it is now possible to directly prepare specimens for the TEM from powder particles using the lift out method. The FIB sample preparation technique of thinning and processing is becoming more mature [16]. However, the excavation depth was limited to approximately 100 *μ*m. The deeper matrix of 100 *μ*m to several hundred micrometers cannot be prepared by FIB upon surface dig and take-off method.

Therefore, surface deformed layers urgently need microstructure characterization, especially on the topmost layer and several hundred micrometers in depth. The sample preparation was the most important part for microstructure study. Some preparation methods for cross-sectional TEM specimen are reported earlier [17, 18]. This study introduced a special sample preparation method—the face-to-face paste method—for the requirements of EM analysis. The feasibility was verified by those samples of the face-to-face paste method for EM observation.

## 2. Experimental Method

**Figure 1:**
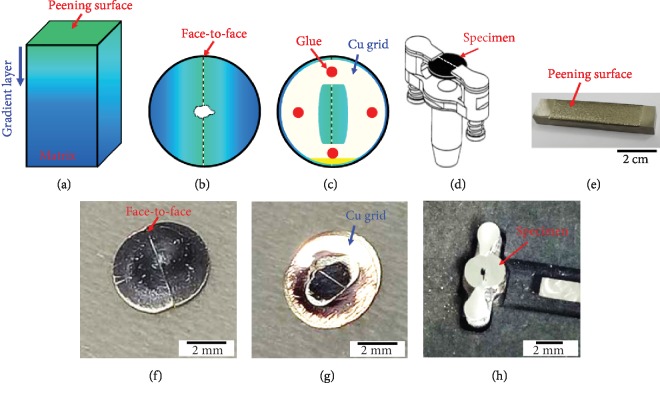
Topmost layer TEM sample preparation of (a–d) schematic diagrams and (e–h) corresponding real sample images. (a, e) Shot-peened specimen, face-to-face pasted sample of (b, f) specimen side and (c, g) Cu grid side, and (d) schematic diagram of TEM specimen in the ion milling holder and (h) final TEM specimen with a hole in the center.

Figures 1(a)–1(d) show the schematic diagram of the topmost layer TEM sample preparation after shot peening. Diagrammatic drawing of the shot-peened specimen with a gradient deformation layer is shown (Figure 1(a)). Owing to the fact that the diameter of TEM specimen is limited in 3 mm, the face-to-face paste method was implemented to piece together a circle with 3 mm diameter. A face-to-face paste method (Figure 1(b)) specimen side and (Figure 1(c)) Cu grid side were well required for electron microscope analysis. Final thinning was used by ion milling; the TEM specimen was fixed in the strut (Figure 1(d)), and finally the center will be thinned out. Corresponding real sample images are shown in Figures 1(e)–1(h).

The pasted glue was G1 glue bought from Gatan official website; detail steps of specimen preparation were through the following:

- Pour the epoxy liquid on the slide glass with the resin to have a harden ratio of 1 : 10
- Use a toothpick to mix them
- Paste it on a standard Cu TEM grid (Figures 1(c) and 1(g) and cure the resin at about 100°C during pasting
- Fix the specimen on the milling holder as shown in Figure 1(d)
- Proceed with grinding, polishing, dimpling, and ion milling
- Figure 1(h) shows the specimen after ion milling

At the final ion milling step, the energy was 3 kV and the milling angle was 4°.

The residual stress was measured by X-ray stress analyzer (LXRD, Proto, Canada) using sin2*ψ* method. The Cr K*α* radiation with a wavelength *λ* = 2.2897 Å was used to determine ferrite (211) diffraction peak. Successive steps of material removal from the impacted surface to ~400 *μ*m depth through electrochemical polishing were conducted in order to perform residual stress measurements in depth. The cross-sectional specimens for the electron back-scattered diffraction (EBSD) analysis were prepared via electrolytic polishing. The top treated surface specimens were thinned via polishing to 90 *μ*m. EBSD specimens were electropolished (Struers TenuPol-5) at room temperature with an electrolyte of 10% perchloric and 90% acetic acid. EBSD measurements were performed with FESEM, JSM-7800F system installed in an oxford scan mode, with a beam step size of 400 nm.

The 34CrMo4 martensitic stainless steel was commercial, which is always used as a drill stem in oil exploration. The chemical composition was consistent with the nominal material element and mainly consisted of weight percent of 0.3-0.37 of C, 0.9-1.2 of Cr, 0.15-0.3 of Mo, 0.6-0.9 of Mn, ≤0.035 of S, ≤0.4 of Si, ≤0.025 of P, and balanced by Fe. Before the shot peening, the specimens are heat treated by quenching from 870°C and annealing in 560°C for 2 h. The description of the shot peening equipment has been reported elsewhere. Prior to the treatment, the plate surface was ground to 2000 grit with SiC metallographic paper. The shot peening processing was carried out for 1, 5, 10, and 15 min, and cast steel shots 0.6 mm in diameter. All thickness and depth in this study are not strictly accurate. It allows for an error of 5%.

## 3. Results and Discussion

**Figure 2:**
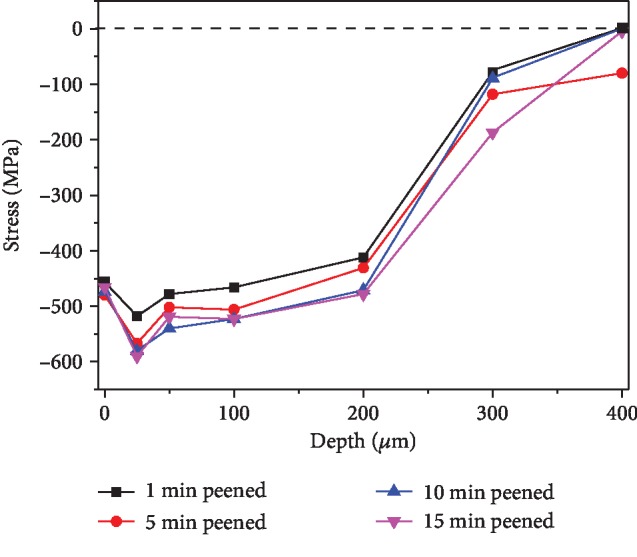
Residual stress analysis of 1~15 min shot-peened specimens.

Figure 2 shows the depth distributions of residual stress after shot peening. With increasing of shot peening intensities, the residual stress will increase from 1 to 5 min. However, it does not increase anymore during peening time from 5 to 15 min. They attain a peak value of -600 MPa at the depth of around 25 *μ*m. The residual stresses return to zero as depth increases to around 400 *μ*m. Therefore, a stronger processing is considered ineffective because of the residual stress of 34CrMo4 steel was considered saturated at 5 min peened.

**Figure 3:**
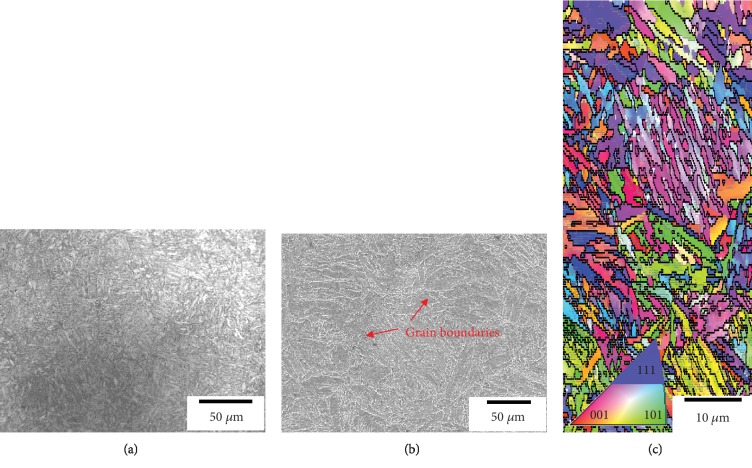
Microstructure characterization of 34CrMo4 Steel (a) OM, (b) SEM, and (c) EBSD mapping.

Microstructure analysis of 1~15 min peened specimens was compared; the details and analysis method will be mainly exhibited by 5 min peened specimen.  Figure 3 shows the microstructure of 34CrMo4 steel matrix: optical microscope (OM) in a wide range view (Figure 3(a)). SEM image shows large grain boundaries, and grain size is ~30 *μ*m (Figure 3(b)). EBSD inverse pole figure mapping shows the lath structures of martensitic steel (Figure 3(c)). The microstructure of the specimen was a typically tempered martensite, with a relatively homogeneous ferrite grain structure.

**Figure 4:**
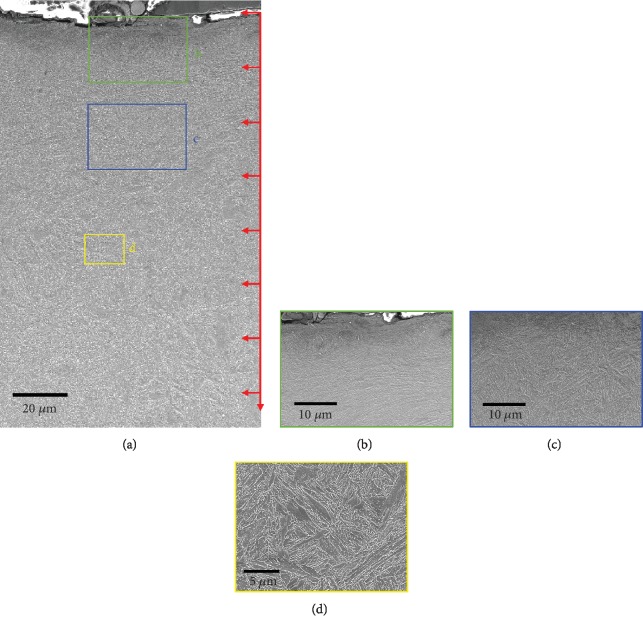
SEM images of the cross-sectional view of martensite steel after shot peening for 5 min (a) and high-magnification images of (b–d) corresponding region marked in (a).

Figure 4 shows SEM images of the 34CrMo4 martensite steel after shot peening for 5 min: cross-sectional view (Figure 4(a)), high magnification images of the deformed structure in the top layer (Figure 4(b)), the gradients structure (Figure 4(c)), and matrix of the steel (Figure 4(d)). The effect depth of deformed structure was 100 *μ*m; grain refinement and deformation were the main features in the layer.

**Figure 5:**
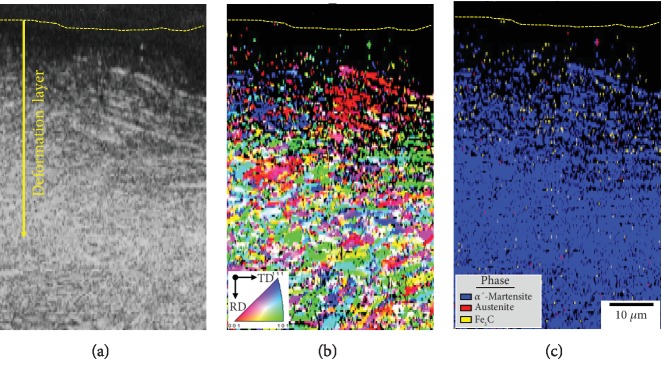
EBSD images of the cross-sectional view of martensite steel after shot peening for 5 min: (a) image quality, (b) inverse pole figure, and (c) phase mapping.

Figure 5 shows the EBSD analysis of image quality (IQ) image (Figure 5(a)), inverse pole figure (IPF) (Figure 5(b)), and phase mapping (Figure 5(c)). The residual stress was rich in the deformation layer after shot peening, which has influenced the EBSD recognition rate. The colors in Figure 5(b) represent different orientations as shown by the standard triangle (inset in Figure 5(b)). The presence of all colors at similar frequencies indicates that the shot-peened specimens fail to exhibit a strong texture. Phase mapping shows that the main phase was *α*′-martensite in body-centered cube (BCC) and bits of austenite and Fe_3_C precipitate. As an additional strategy, the precipitated phase must be Fe_3_C from the study of iron-carbon phase diagram [19].

**Figure 6:**
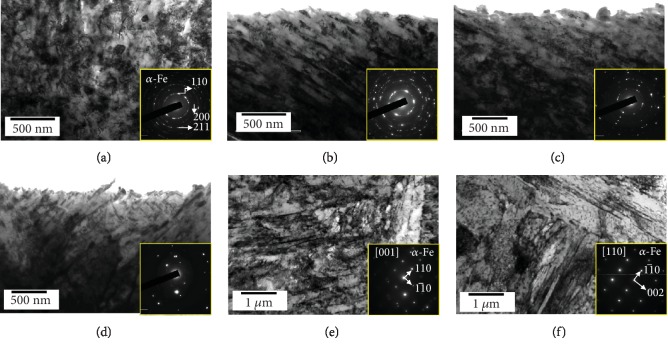
TEM analysis of the 34CrMo4 martensite steel after shot peening for 5 min: (a) topmost layer, (b) 50 *μ*m depth, (c) 100 *μ*m depth, (d) 200 *μ*m depth, (e) 400 *μ*m depth form top surface, and (f) matrix before shot peening. Corresponding SADPs are inside.

Figure 6 is TEM characterization of the topmost layer on the deformed layers. At the topmost layer (Figure 6(a)), the nanosized grains were obvious by high-density dislocation entanglement, which corresponds to the SADP showing a ring pattern taken on this area. At the depth of 50 *μ*m (Figure 6(b)), high-density nanothickness lath structure was obvious. Some dislocation cell and new grain boundaries are visible in the 100 *μ*m depth region, as shown in Figure 6(c). In the 200 *μ*m depth region (Figure 6(d)), the dislocation and subgrains had sharp boundaries. At the 400 *μ*m depth (Figure 6(e)), original grains are seen the same as in the matrix (Figure 6(f)) typically tempered martensite. The microstructural evolution from original grains to nanograins was apparent in the TEM analysis by the decreasing of depth.

**Figure 7:**
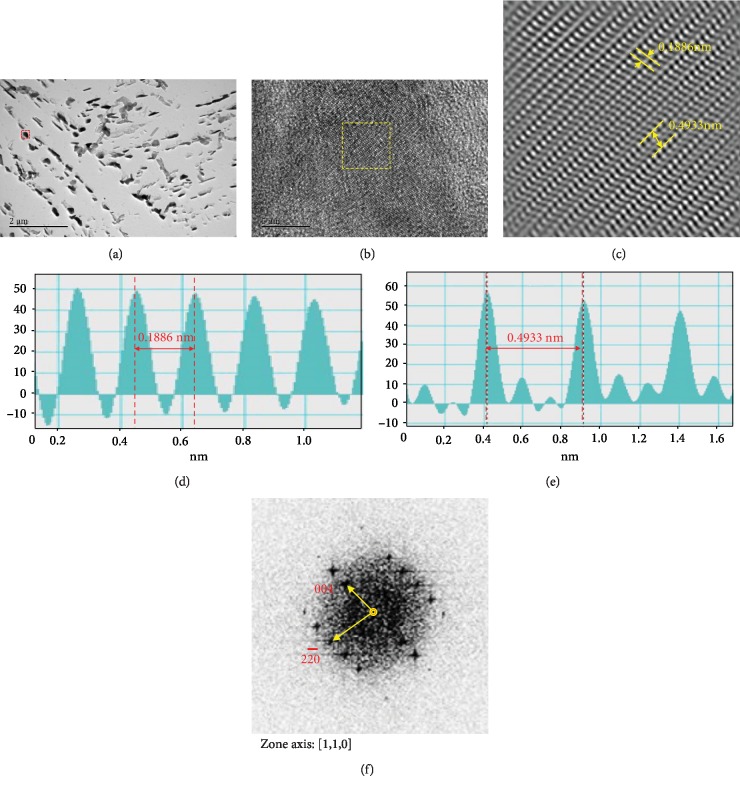
TEM analysis of the martensite steel: (a) TEM image on precipitates, (b) high-magnification TEM images, (c) iFFT image, (d, e) measurement of the distance of atom plans, and (f) index of electron diffraction pattern form the yellow square in (b).

Figure 7 shows the TEM analysis of precipitates; the TEM sample preparation is by carbon extraction replica technology [20]. High-magnification TEM images in Figure 7(b), iFFT image (Figure 7(c)), measurement of the distance of atom plans (Figures 7(d) and 7(e)), and index of electron diffraction pattern (Figure 7(f)) form the yellow square in Figure 7(b). Compared to standard PCPDF, card # 030411 [21] was well matched.

**Figure 8:**
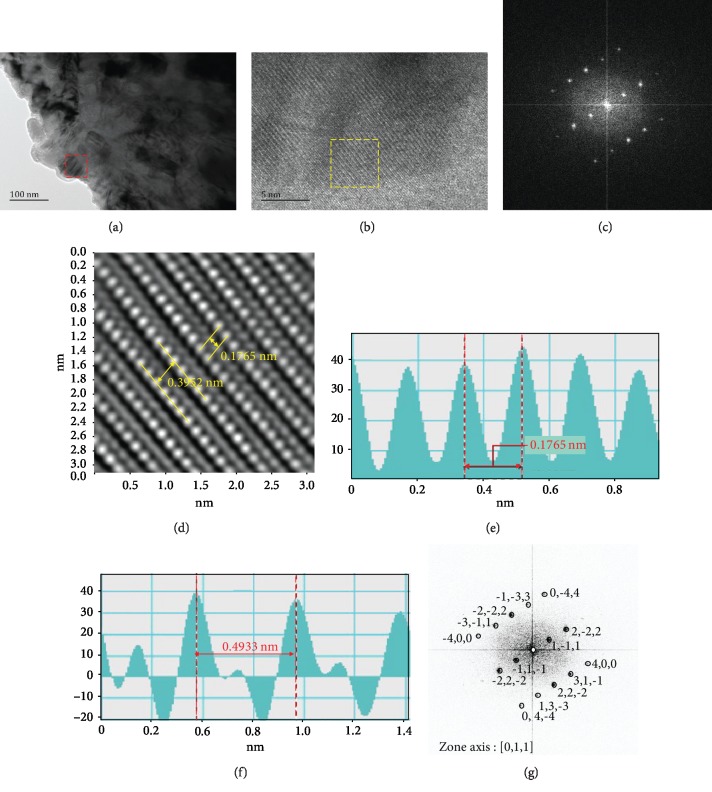
TEM analysis of the martensite steel after shot peening: (a) TEM image on the topmost layer, (b) high-magnification TEM images, (c) FFT and (d) iFFT images, (e) and (f) measurement of the distance of atom plans, and (g) index of electron diffraction pattern in (c).

Figure 8 shows the TEM and high-resolution TEM (HRTEM) analysis of the TEM analysis of the martensite steel after shot peening: TEM image on the topmost layer (Figure 8(a)) and HRTEM images (Figure 8(b)) taken in the red dotted box in Figure 8(a). In the software of digital micrograph, selected squares marked in Figure 8(b) can get the fast Fourier transformation (FFT) image see in Figure 8(c). Apply mask and then get inverse FFT image in Figures 8(d)–8(f) measurement of the distance of atom plans. Compared to standard PCPDF, card # 882324 [22] was well matched. The lattice of FCC, space group of Fm$\bar{3}$m(225), *a* = 0.343 nm, *d*_111_ = 0.1980 nm, *d*_200_ = 0.1715 nm, and *d* = 0.3952 nm were twice as the distance of the *d*_111_, and zone axis was [0,1,1] in Figure 8(g). The FCC crystal was discovered in the topmost layer of martensite steel (BCC) after shot peening. In the phase mapping by EBSD (Figure 5(c)), FCC crystal was identified in red color near the topmost layer. Phase transformation from FCC to BCC was reported in the study of 301 austenite stainless steel upon ultrasonic shot peening in preliminary [5]. The reverse phase transformation from BCC to FCC was discovered in this study. However, the dynamic phase transformation between FCC and BCC upon severe plastic deformation needs substantial evidence in further research.

## 4. Summary and Conclusion

The main works are residual stress test and microstructure characterization of 34CrMo4 steel upon various shot peening treatments. Following are the findings:

1. The residual stress distribution with effect depth was found increasing upon the shot peening. The residual stress of 5 min shot-peened specimen was saturated. The maximum compressive stress value is -600 MPa at the depth of 25 *μ*m
2. Microstructure evolution characterization was analyzed by various electron microscope technologies. Nanograins were observed on the top most peening layer. In nanothickness lath structure and nanosized grains, precipitates were in gradient deformation layer. Grain refinement was clearly observed in the gradient deformation layer
3. The FCC crystal was discovered in the topmost layer of BCC after shot peening. The dynamic phase transformation between FCC and BCC upon shot peening needs substantial evidence in further research

## Data Availability

No data were used to support this study.
